# Eco-evolutionary responses of *Bromus tectorum* to climate change: implications for biological invasions

**DOI:** 10.1002/ece3.542

**Published:** 2013-04-12

**Authors:** Tamara J Zelikova, Ruth A Hufbauer, Sasha C Reed, Timothy Wertin, Christa Fettig, Jayne Belnap

**Affiliations:** 1 Southwest Biological Science Center, U.S. Geological SurveyMoab, Utah, 84532; 2 Botany Department, Berry Biodiversity Conservation Center, University of WyomingLaramie, Wyoming, 82071; 3 Department of Bioagricultural Sciences and Pest Management and Graduate Degree Program in Ecology, Colorado State UniversityFort Collins, Colorado, 80523; 4 Institute of Genomic Biology, University of IllinoisUrbana, Illinois, 61801

**Keywords:** *Bromus tectorum*, cheatgrass, climate change, Colorado Plateau, phenology

## Abstract

How plant populations, communities, and ecosystems respond to climate change is a critical focus in ecology today. The responses of introduced species may be especially rapid. Current models that incorporate temperature and precipitation suggest that future *Bromus tectorum* invasion risk is low for the Colorado Plateau. With a field warming experiment at two sites in southeastern Utah, we tested this prediction over 4 years, measuring *B. tectorum* phenology, biomass, and reproduction. In a complimentary greenhouse study, we assessed whether changes in field *B. tectorum* biomass and reproductive output influence offspring performance. We found that following a wet winter and early spring, the timing of spring growth initiation, flowering, and summer senescence all advanced in warmed plots at both field sites and the shift in phenology was progressively larger with greater warming. Earlier green-up and development was associated with increases in *B. tectorum* biomass and reproductive output, likely due early spring growth, when soil moisture was not limiting, and a lengthened growing season. Seeds collected from plants grown in warmed plots had higher biomass and germination rates and lower mortality than seeds from ambient plots. However, in the following two dry years, we observed no differences in phenology between warmed and ambient plots. In addition, warming had a generally negative effect on *B. tectorum* biomass and reproduction in dry years and this negative effect was significant in the plots that received the highest warming treatment. In contrast to models that predict negative responses of *B. tectorum* to warmer climate on the Colorado Plateau, the effects of warming were more nuanced, relied on background climate, and differed between the two field sites. Our results highlight the importance of considering the interacting effects of temperature, precipitation, and site-specific characteristics such as soil texture, on plant demography and have direct implications for *B. tectorum* invasion dynamics on the Colorado Plateau.

## Introduction

Climate change is one of the most critical environmental challenges of the 21st century and thus a central focus in ecology today lies in understanding how populations, communities, and ecosystems will respond to the environmental changes they face. In terrestrial ecosystems, the responses of plants, the primary producers, lay the foundation for all other members of an ecosystem. Characterizing how individual plant species respond to change is an important goal for researchers and land managers alike. This is especially important when considering the differences between responses of native versus invasive species (Dukes and Mooney 1999; Weltzin et al. 2003; Moore 2004; Thuiller et al. 2005). Several lines of evidence suggest that climate change may benefit nonnative plants more than native plants (Smith et al. 2000; Zinka 2003; Hellmann et al. 2008; Walther et al. 2009; Bradley et al. 2010; Zhang et al. 2011). In addition, long-term trends show native perennial grasses in arid ecosystems decrease in cover in warm and dry years (Munson et al. 2011). Coupled with decreases in the cover of native grasses and shrubs, the abundance, range, and proportion of nonnative grasses is expected to increase as the climate continues to change (Archer and Predick 2008; Zhang et al. 2011).

Phenology is an essential metric for tracking and quantifying the impacts of climate change because the timing of important phenological events, such as flowering and fruiting in plants, is sensitive to climatic cues (Cleland et al. 2007; Sherry et al. 2007), temperature, water availability, and photoperiod (Partanen et al. 1998; Badeck et al. 2004). Changes in plant phenology can have far-reaching consequences, from altering species interactions and community dynamics (Parmesan 2006; Cleland et al. 2007) to driving the annual cycle in carbon uptake (Keeling et al. 1996; Myneni et al. 1997). Recent meta-analyses of long-term observational data report that the onset of spring has advanced at an average of 2.5 days per decade (Parmesan and Yohe 2003; Menzel et al. 2006) and plants that flower in the spring and early summer on average show the greatest shift in development, possibly because their phenology is linked to snowmelt (Sherry et al. 2007). Experimental warming studies yield similar results, showing increased growth and extended duration of reproductive phases for many species (Sherry et al. 2007; Wolkovich et al. 2012), though there is evidence to suggest that experiments under-predict phenological responses to warming (Wolkovich et al. 2012).

*Bromus tectorum*, an invasive annual grass from Eurasia and North Africa (Mack 1981), has spread throughout the western United States after initial introduction around 1880 and is now a species of major management concern (Di'Tomaso 2000). Climatic conditions may influence the establishment of *B. tectorum* (Bradford and Lauenroth 2006), and current predictive models suggest that summer and spring precipitation and winter maximum temperature determine the distribution of *B. tectorum* in western United States, predicting a contraction of its southern range under climate change scenarios and indicating low invasion risk for the Colorado Plateau in southeastern Utah (Bradley 2009; Bradley et al. 2009). Current models project temperatures at lower elevations within the Colorado Plateau to rise by 4–6°C by 2100 (Seager et al. 2007). There is more uncertainty associated with projections of future precipitation regimes for the desert southwest, including for the Colorado Plateau (Smith et al. 2005). However, shifts in precipitation patterns, when coupled with increasing temperatures, will likely lead to an overall transition to a much drier climate across the Colorado Plateau (NAST 2000, Seager et al. 2007; Cayan et al. 2010; Seager and Vecchi 2010). Predictive models, such as those used by Bradley and colleagues (2009), rely on changes in annual temperature and precipitation, though the timing of precipitation may be more important in arid regions such as the Colorado Plateau (Lin et al. 1996; Schwinning et al. 2005a,b; Schwinning et al. 2008). We tested the prediction that *B. tectorum* is expected to respond negatively to warmer climate on the Colorado Plateau (Bradley et al. 2009) in a 4-year field warming experiment, superimposing an experimental manipulation upon a natural variation in climate. We assessed how phenology, biomass, and reproduction of *B. tectorum* respond to temperature increases on the Colorado Plateau. In contrast to a model predicting range contraction with warming (Bradley 2009; Bradley et al. 2009), we predicted that warming would accelerate phenological development in this annual invasive grass when soil moisture was adequate and thereby result in increased biomass and annual reproductive output. In a complimentary greenhouse study, we assessed whether warming treatments and changes in *B. tectorum* biomass and reproductive output influenced offspring performance and examined genetic variation in a subset of offspring from warmed plants for evidence of evolutionary adaptation to warming.

## Materials and Methods

### Experimental design

**Figure 1:**
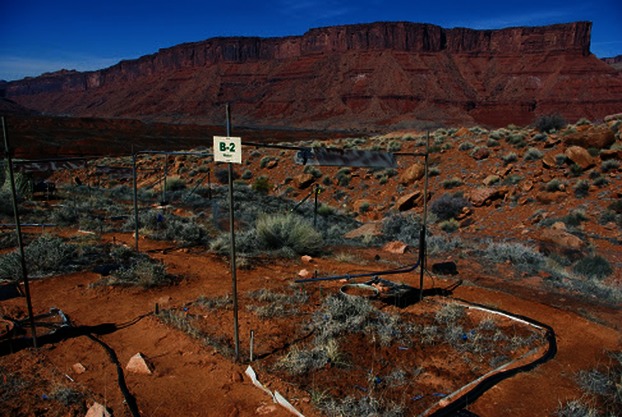
Photograph of an experimental plot at the Castle Valley field site.

**Figure 2:**
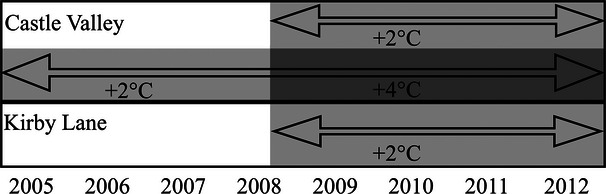
Experimental design at two sites, Castle Valley and Kirby Lane. The Castle Valley site was established in 2005, with 2°C warming applied to five replicate plots and associated nonwarmed controls. The 2°C warming plots were converted to 4°C in 2009 and an addition set of plots was established at Castle Valley, with 2°C warming applied to five replicate plots, with five associated nonwarmed controls. The Kirby Lane experimental site was also established, with 2°C warming treatments applied in five replicates and five nonwarmed controls in 2009.

The experiment was initiated in the Fall of 2005 on the Upper Colorado Plateau and from 2005 to 2008, the study focused on measuring the effects of the climate change manipulations on the structure and function of biological soil crust communities (Zelikova et al. 2012). The Colorado Plateau region is generally characterized as a cool desert ecosystem, receiving approximately 65% of the annual precipitation in winter months. We established two field sites near Moab, UT: Castle Valley, UT (38.67485N, −109.4163 W, 1310 m elevation; hereafter “Castle Valley”, Fig. 1), initiated in 2005, and a site within the Moab valley approximately 35 km from the Castle Valley site (elevation 1226 m; hereafter “Kirby Lane”), initiated in 2009. The soils at Castle Valley are classified as sandy loam, calcereous, Rizno series and the vegetation is dominated by two perennial grasses, *Pleuraphis jamesii*, *Stipa (Achnatherum) hymenoides*, an annual invasive grass *B. tectorum*, and the shrub *Atriplex confertifolia*. The Castle Valley site aspect is west, with the +4°C block of plots located on a 10 degree slope and the +2°C block is located on a 13 degree slope. Kirby Lane soils are classified as sandy and vegetation is dominated by two grasses, *S. hymenoides* and *B. tectorum*. The Kirby Lane site aspect is south and it is relatively flat. Warming treatments at these sites were applied to 2 × 2.5 m plots in replicates of five in a randomized block design and were paired with controls. From 2005 to 2008, the soil surface in Castle Valley plots was warmed +2°C above ambient (Fig. 2). During this time, a new climate model (Seager et al. 2007) predicted a larger temperature increase for the southwest region. To more accurately reflect this prediction, the original +2°C plots were increased to +4°C warming beginning in January 2009. Additionally, five blocks of +2°C warmed and control plots (a total of 10 plots) were added in randomized block design at the Castle Valley site. The Kirby Lane site was added in January 2009 with the installation of five blocks of +2°C warming treatments paired with controls, following the same design as those in Castle Valley (Fig. 2).

The warming treatments were delivered by one (to achieve +2°C warming) or two (to achieve +4°C warming) 800 W infrared radiant heaters (Kalglo Model MRM-1208, with modified reflectors, see Harte et al. 1995) installed at 1.3 m above the surface of the plots and all lamps were oriented in a north-south direction in relation to the plots to minimize shading of the plot (Harte et al. 1995; Kimball 2005). Each lamp emits an average of 60 Wm^−2^ of heat, equivalent to +2°C. The target soil warming was achieved during night-time, while daytime warming was slightly more seasonally variable. The control plots each have dummy lamps of the same size and shape, but these do not emit a heating treatment. Leaf temperatures of plants under the lamps were monitored with radiometers to ensure they did not exceed the warming target. Soil temperatures were monitored with three-tipped thermopile probes at 2, 5, and 15 cm depth at Castle Valley and 5, 10, and 20 cm depth at Kirby Lane to assure that the target warming was achieved. A meteorological station at each site recorded air temperature and relative humidity, precipitation, and wind speeds.

### Phenology

Phenology measurements were initiated in 2009. We tracked the timing and duration of phenophases throughout the growing season following a modified field observational protocol based on Wein and West (1971). We established five permanent 15 × 15 cm subplots within each plot and each week, starting in April in 2009 and February in 2010 and 2011, plants were scored based on their phenological stage. In 2009, we began field observations in April, after spring green-up had occurred. *B. tectorum* plants were scored on a plot-basis, with each plot given a score based on the stage at which the majority of the individual plants within that plot were found in. In 2010 and 2011, we individually scored each 15 × 15 cm subplot. The four primary developmental stages were as follows: (1) spring growth initiation, (2) anthesis, the onset of flower production followed by the onset of seed production, (3) maturation, when the growth rate slows and seeds begin to drop off and disseminate, and (4) senescence, when over 90% of the aboveground plant tissue experiences loss of green coloration. We recorded the Julian day when the transition from one phenophase to the subsequent phenophase occurred. In 2012, a different field protocol was used to measure phenology and those results are not presented here.

### Biomass and reproductive output

Because we could not destructively harvest the plants we were monitoring within the long-term experimental plots, we developed site-specific allometric equations for estimating biomass annually, using plants outside the experimental plots. Stalk height multiplied by the number of individuals within the 15 × 15 cm subplots was the best predictor of *B. tectorum* biomass, explaining 68% of the variation in biomass. Thus, for biomass estimates, we counted all plants and measured the height of 10 individuals per subplot. To estimate reproductive output, we counted the number of spikelets produced per inflorescence in the subplots.

### Greenhouse study

In 2010, we collected three *B. tectorum* individuals with inflorescences from each of the five replicate experimental plots and five associated ambient plots across both experimental sites, taking care not to remove plants from inside the permanent subplots. In 2011, we collected 5 spikelets from 10 individuals per plot. Because direct seed counts require destructive dissection of each floret inside the spikelet (McKone 1985), we estimate reproductive output by counting spikelets rather than direct seed production. Each year, we also collected 15 *B. tectorum* plants from each research site to represent the background wild population (WP). All plant material was stored in manila envelopes at room temperature for 5 months until germination trials (Allen and Meyer 2002; Roundy et al. 2007). To measure seed viability, we conducted germination trials by placing 3–5 spikelets with multiple florets per spikelet from each parental source on wet filter paper (Whatman #5, 90 mm) inside Petri dishes. We added fungicide and 10% bleach solution to each plate and vernalized the seeds at 2°C with a 12 h light/12 h dark cycle for 10 weeks. Germination was monitored weekly and a floret was considered germinated upon radicle emergence. Seedlings were transplanted into individual containers filled with 50:50 mixture of standard potting mix and sterile sand. Following a 10-week vernalization, all seedlings were moved into the greenhouse and watered once per week. Seedling phenology was recorded weekly on an individual plant basis, tracking all individuals in 2011 and one individual per parental source in 2012. Plants were harvested upon reaching the seed scatter phenophase and we separated inflorescences, aboveground, and belowground biomass. Roots were washed with distilled water to remove remaining soil particles. All biomass was dried at 60°C for 24 h and weighed. Parental source spikelets and offspring leaves and spikelets from the 2011 greenhouse populations were analyzed for total C and nitrogen (N) content with a CHN analyzer (Costech Analytical Technologies, Inc., Valencia, CA).

### Microsatellite variation

Given that *B. tectorum*, is a cleistogamous species that only rarely shows evidence of outcrossing (Kao et al. 2008), it is important to determine whether the research plots contained essentially the same genotypes, or if they are genetically diverse. For a first glance into diversity at the site, we genotyped 40 individuals (20 offspring of plants from the +4°C warmed plots and 20 offspring of plants from ambient plots from the Castle Valley field site) at 5 microsatellite loci (Loci 4, 5, 26, 30, 33 from Ramakrishnan et al. 2002) following the methods in Kao et al. (2008).

### Analyses

All statistical analyses were performed using JMP 10.0 software (SAS Institute, Cary, NC). To examine the effects of field warming treatments on plant density, biomass, and reproductive output, we used a two-way ANOVA, with treatment as the main effect and block as a random effect. The effect of warming on phenology was analyzed using a survival analysis, with the Julian day a particular phenophase was reached as a response variable and the proportion of the population reaching that phase as the survival function. This analysis includes a censoring factor and allows for the inclusion of data points even when a particular phenophase is not reached. The results of the Wilcoxon nonparametric test are reported in Table 2. In addition, we calculated the effect size of warming as the difference between warmed and ambient plots and compared that difference against a hypothesized different of zero with a one-sample *t*-test. Because both temperature and precipitation varied substantially between sampling years and *B. tectorum* is an annual grass, data for each year were analyzed separately. Due to substantial differences in soil properties and elevation between the Castle Valley and Kirby Lane sites, analyses were also performed separately for each site. Total C and N concentrations and C:N ratios were compared between warmed and ambient seed sources using a Student's *t*-test, with separate analyses for field-collected seeds, F1 aboveground biomass, and F1 seeds. Germination rate was calculated as the number of seedlings that germinated per spikelet and survival was calculated as the number of plants that survived until harvest divided by the total number of transplanted seedlings. Because we do not have a direct estimate of the number of seeds per spikelet, germination rate may confound the effects of warming treatments on seed and spikelet production such that increased viability of seeds from warmed plants may alternatively indicate increased seed production in warmed plants. We averaged across replicate seedlings from the same seed source and used a one-way ANOVA to compare between warmed and unwarmed plots, using a Tukey–Kramer post hoc analysis to differentiate between different groups. We used GenoDive (Meirmans and Van Tienderen 2004) to calculate heterozygosity and average number of alleles across loci, and effective number of alleles between warmed and ambient plots. Additionally, we tallied the distinct multilocus genotypes present at the site.

## Results

**Figure 3:**
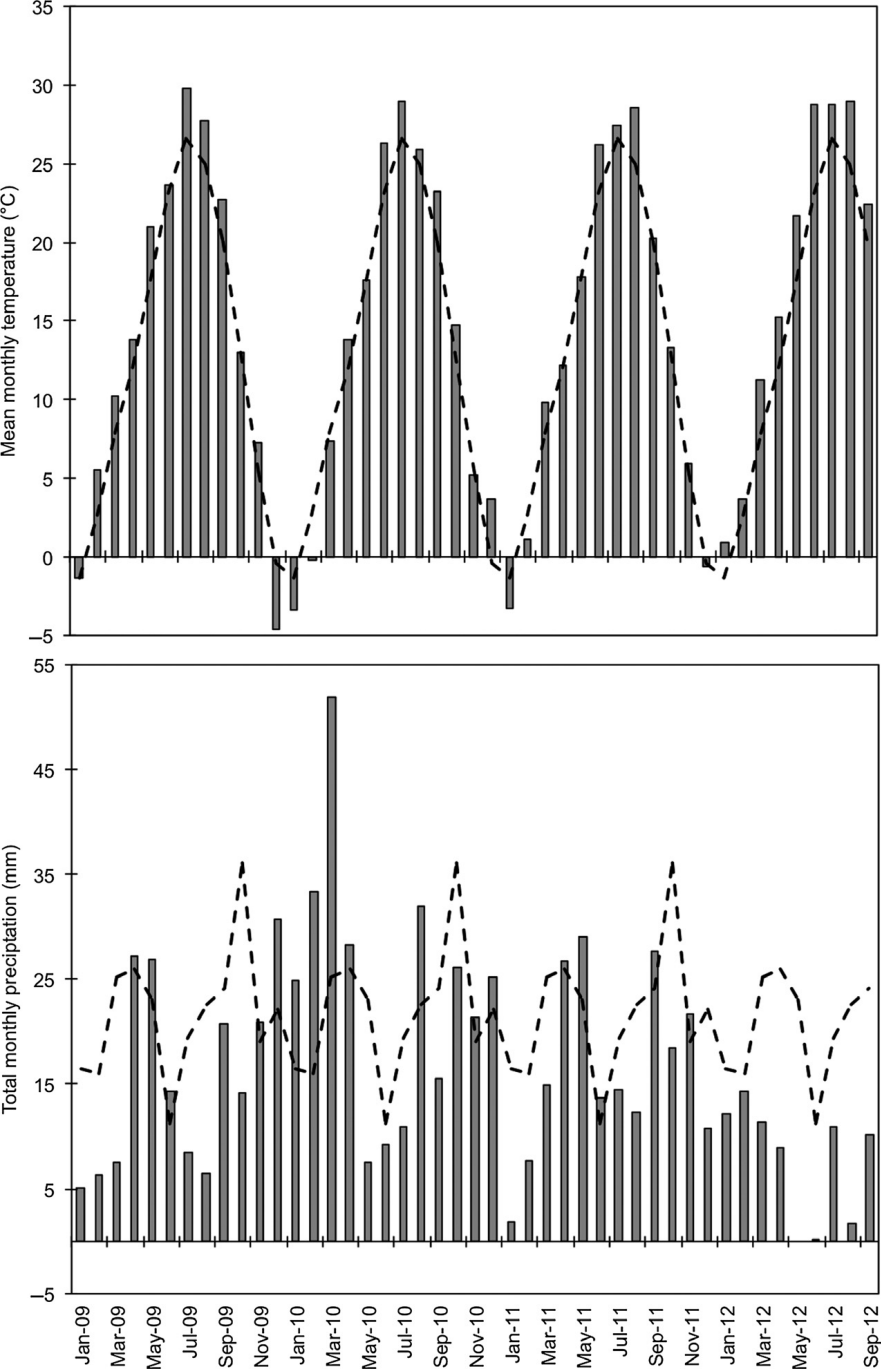
Average monthly temperature and total monthly precipitation for 2009, 2010, 2011, and through August 2012 (filled in bars) relative to the 30 year running average in Castle Valley, UT (dashed lines) Data source: Western Regional Climate Center (http://www.wrcc.dri.edu/cgi-bin/cliMAIN.pl?ut1241)

The experimental sites experienced above-average monthly temperatures for the majority of 2009–2012, compared with the 30-year mean from 1982 to 2012 (Fig. 3). Annual precipitation at this site was below the 30-year mean in 2009, 2011, and through September 2012 and wintertime (October–February) and spring growth (March–May) precipitation were above the 30-year mean only in 2010 (Table 1).

**Table 1:** Annual temperature and precipitation data collected in Castle Valley from 2009 through September 2012

	30 year mean	2009	2010	2011	2012
Mean annual temperature (°C)	12.7	14.1	13.6	13.2	15.3
Annual precipitation (mm)	261.7	189	286.4	199.1	116.5
October–February precipitation (mm)	110	71	124	82	87
March–May precipitation (mm)	74	62	88	71	20
June–September precipitation (mm)	77	50	68	58	39

Wintertime precipitation includes total precipitation from October through February, *B. tectorum* growing season represents total precipitation from March through May, and summertime precipitation from June through September. The 30 year mean annual temperature and precipitation data source is the Western Regional Climate Center http://www.wrcc.dri.edu/cgi-bin/cliMAIN.pl?ut1241).

### Phenology

In Castle Valley in 2009, the onset of flowering did not differ between warmed and ambient plots, regardless of warming level (Table 2). Similarly, the timing senescence also did not differ between warmed and ambient plots. At Kirby Lane, *B. tectorum* flowered, scattered seeds, or senesced at the same time in warmed and ambient plots, and the entire life cycle was initiated and completed earlier at Kirby Lane than at Castle Valley.

**Table 2:** Statistical results comparing the timing (DOY) of green-up, flowering, and senescence phenophases between warming treatments and their associated controls in Castle Valley and Kirby Lane experimental sites

Site	Year	Phenophase	+4°C		+2°C	
	
			Wilcoxon χ^2^	*P*	Wilcoxon χ^2^	*P*
Castle Valley	2009	Flowering	1.5	0.22	2.18	0.14
		Senescence	2.25	0.32	0.63	0.43
	2010	Green-up	30.1	**<0.0001**	7.3	**0.0069**
		Flowering	18.49	**<0.0001**	5.57	**0.02**
		Senescence	22.93	**<0.0001**	3.03	0.08
	2011	Green-up	0.0074	0.93	0.48	0.48
		Flowering	1.2	0.27	0.16	0.69
		Senescence	0.007	0.93	0.07	0.78
Kirby Lane	2010	Green-up			0.16	0.69
		Flowering			6.57	**0.01**
		Senescence			3.03	0.08
	2011	Green-up			1.75	0.19
		Flowering			10.8	**0.001**
		Senescence			0.8	0.37

There was no variation in the timing of flowering or senescence in Kirby Lane in 2009 and those analyses are not presented.

Values in bold indicate statistically significant differences.

**Figure 4:**
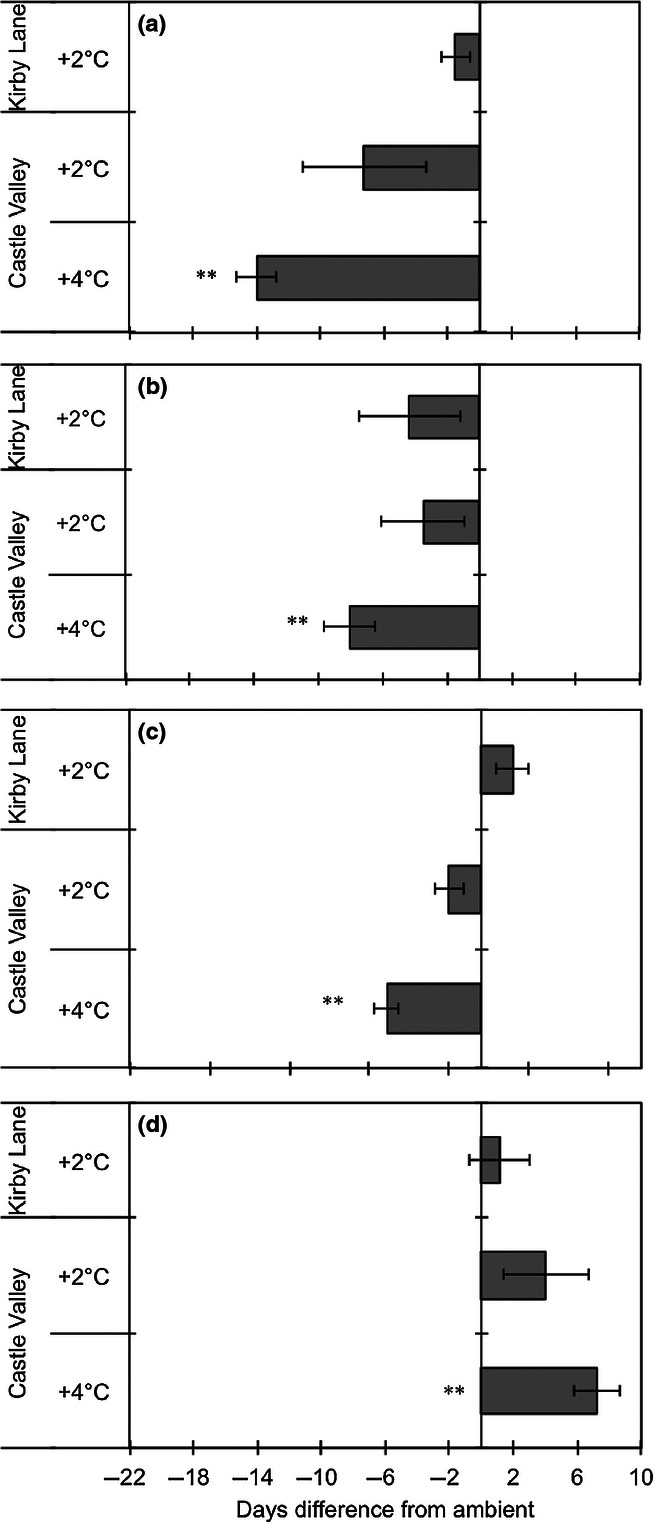
Differences in day of year (DOY) of (a) spring growth initiation, (b) flowering, (c) senescence, and (d) length of growing season between warmed and ambient plots in 2010. Means (±SE) were calculated by subtracting the DOY when a particular phenophase was reached in the ambient plot from the warmed plot and **the difference between the warmed and ambient plots is significantly different from zero at α = 0.01.

In 2010, a wet winter/spring (Table 1), warming treatments resulted in a substantial shift in *B. tectorum* phenology (Fig. 4). Spring green-up advanced on average 2 weeks earlier in +4°C plots (14 ± 1.3 days) and 1 week earlier in +2°C plots (7 ± 2.1 days), relative to ambient plots in Castle Valley (Fig. 4a). Similarly, the onset of flowering was advanced in +4°C plots (8.1 ± 1.1 days) and +2°C plots (3.5 ± 0.8 days; Fig. 4b). Summer senescence also occurred 5 days earlier in +4°C plots (5.8 ±0.7 days) and by 2 days in +2°C plots (Fig. 4c). Overall, the growing season was extended by 7–10 days in +4°C plots (7.2 ± 1.4 days) and 2–4 days in +2°C plots (4.1 ± 2.7 days; Fig. 4d). At Kirby Lane, spring green-up occurred at the same time in warmed and ambient plots, but the onset of flowering was significantly earlier in the warmed plots (4.4 ± 3.2 days), though all plants senesced in the same week. As a result, the length of the growing season remained unchanged in warmed plots. In 2011, there were no significant effects of warming on any measures of phenology in Castle Valley, but at Kirby Lane, the onset of flowering was advanced by 3.5 days in the warmed plots, though these changes did not alter the length of the growing season.

### Vegetative response

**Figure 5:**
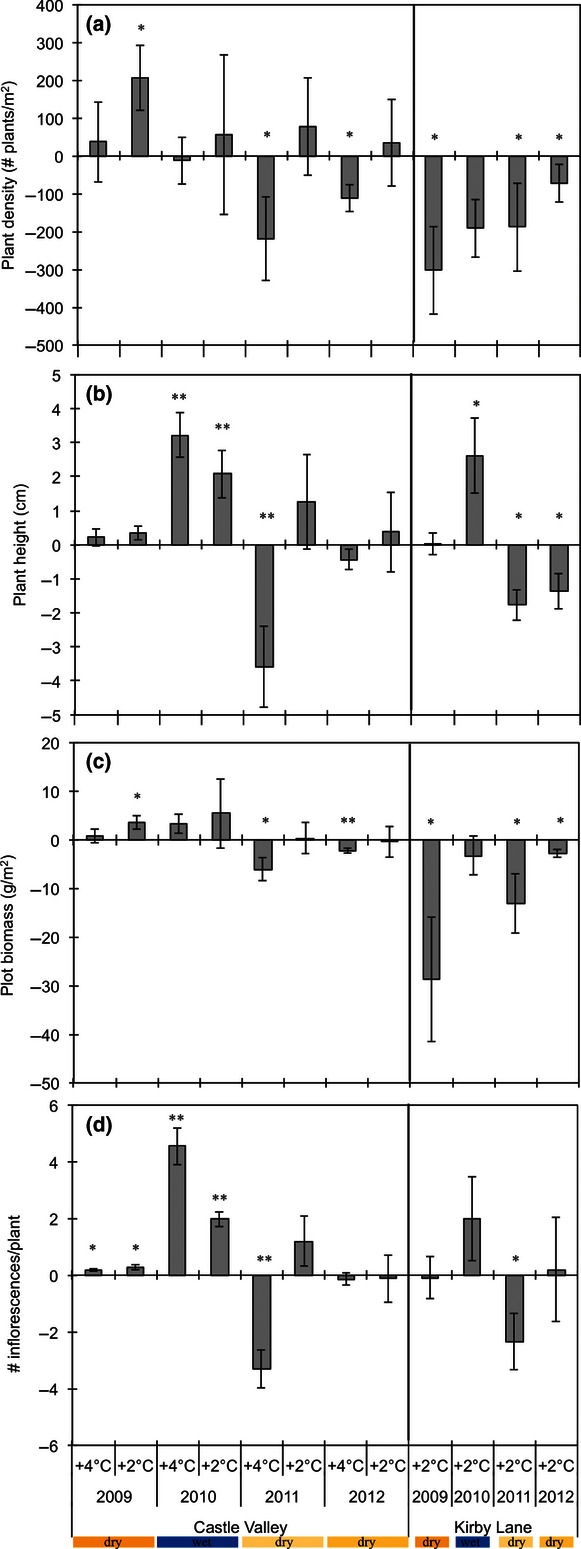
Differences in (a) plant density, (b) plant height, (c) plot biomass, and (d) reproductive output between warmed and ambient plots. Means (±SE) were calculated by subtracting the ambient plot means from the warmed plot means and indicates when the difference between the warmed and ambient plots is significantly different from zero. * difference at α ≥ 0.05, ** difference at α ≥ 0.01.

In 2009, *B. tectorum* density was almost 2× greater in the +2°C plots at Castle Valley (Fig. 5a; Table 3), but did not differ between +4°C and ambient plots. In contrast, plant density was greater in the ambient plots at Kirby Lane. We observed no warming effect on plant density in Castle Valley in the wet year 2010, but density was almost 2× greater in the ambient plots at Kirby Lane. Variation in plant density among the ambient plots was higher in the following dry years 2011 and 2012, in contrast to the variation among the warmed plots. For example, plant density in 2012 varied between 27–613 plants/m^2^ in ambient plots in Castle Valley and 97–800 plants/m^2^ at Kirby Lane. In 2011, plant density was on average more than 2× greater in ambient plots than +4°C plots, but similar between the +2°C and ambient plots at both sites. In 2012, plant density was significantly higher in ambient plots, relative to +4°C plots, but we observed no effect of +2°C warming on plant density at either site (Fig. 5a).

**Table 3:** Statistical results comparing *Bromus tectorum* plant density (# plants/m^2^), height (cm), biomass (g), and reproductive output (# inflorescences/plant) between warming treatments and their associated controls in Castle Valley and Kirby Lane experimental sites

Site	Year	Phenophase	+4°C		+2°C	
	
			*F*	*P*	*F*	*P*
Castle Valley	2009	Plant density	0.08	0.79	5.42	**0.05**
		Height	0.85	0.38	1.36	0.28
		Biomass	0.25	0.63	6.8	**0.03**
		Reproduction	15.4	**0.004**	10.9	**0.01**
	2010	Plant density	0.02	0.88	0.11	0.75
		Height	26.2	**0.009**	12.7	**0.007**
		Biomass	1.43	0.27	0.83	0.39
		Reproduction	54.98	**<0.0001**	3.6	**0.006**
	2011	Plant density	4.6	0.06	0.27	0.62
		Height	14.61	**0.005**	1.88	0.18
		Biomass	7.94	**0.02**	0.009	0.93
		Reproduction	36.24	**0.0003**	0.56	0.48
	2012	Plant density	10.4	**0.03**	0.1	0.76
		Height	0.9	0.35	2.54	0.12
		Biomass	16.34	**0.004**	0.26	0.63
		Reproduction	0.41	0.54	0.21	0.66
Kirby Lane	2009	Plant density			2.77	0.13
		Height			0.01	0.91
		Biomass			1.96	0.2
		Reproduction			0.01	0.91
	2010	Plant density			3.01	0.12
		Height			8.84	**0.02**
		Biomass			0.48	0.51
		Reproduction			2.87	0.13
	2011	Plant density			1.94	0.2
		Height			9.66	**0.004**
		Biomass			3.4	0.1
		Reproduction			7.23	**0.03**
	2012	Plant density			1.64	0.24
		Height			12.97	**0.001**
		Biomass			4.58	0.06
		Reproduction			3.64	0.09

Values in bold indicate statistically significant differences.

Plant height did not differ between warmed and ambient plots at either site in 2009, though plants were substantially taller at Kirby Lane than in Castle Valley. However, in 2010, plants were almost 2× taller in +4°C plots, 1.5× taller in +2°C plots in Castle Valley, and 1.5× taller in the warmed plots at Kirby Lane (Fig. 5b). This trend was reversed in 2011, when +4°C warming had a negative effect on plant height at Castle Valley and +2°C warming had a similar negative effect at Kirby Lane. There were no differences in plant height between warmed and ambient plots in Castle Valley in 2012 (Fig. 5b), but plants were shorter in warmed plots at Kirby Lane in 2012.

In 2009, plant biomass, calculated by multiplying plot plant density by average plant height within the same plot, did not differ between warmed and ambient plots (Fig. 5c). However, plant biomass was greater across all warmed plots in 2010. In 2011, plant biomass remained similar to 2010 in ambient plots in Castle Valley, but was significantly lower in +4°C plots in Castle Valley and +2°C at Kirby Lane, largely driven by changes in plant height rather than changes in plant density. We observed a similar reduction is biomass at Kirby Lane in 2012 (Fig. 5c).

### Reproductive response

Plant reproductive output, measured as the number of spikelets produced per plant, was significantly greater in warmed plots in Castle Valley in 2009 and 2010. While +2°C warming slightly increased reproductive output in Castle Valley in 2011, +4°C warming substantially reduced the number of inflorescences produced per plant. We observed no effect of +4°C or +2°C (Fig. 5d) warming on reproductive output in 2012, when reproductive output was generally low across all plots. At Kirby Lane, reproductive output did not differ between warmed and ambient plots in 2009 and 2012. However, there was a positive effect of warming in 2010 and a negative effect in 2011 (Fig. 5d).

**Figure 6:**
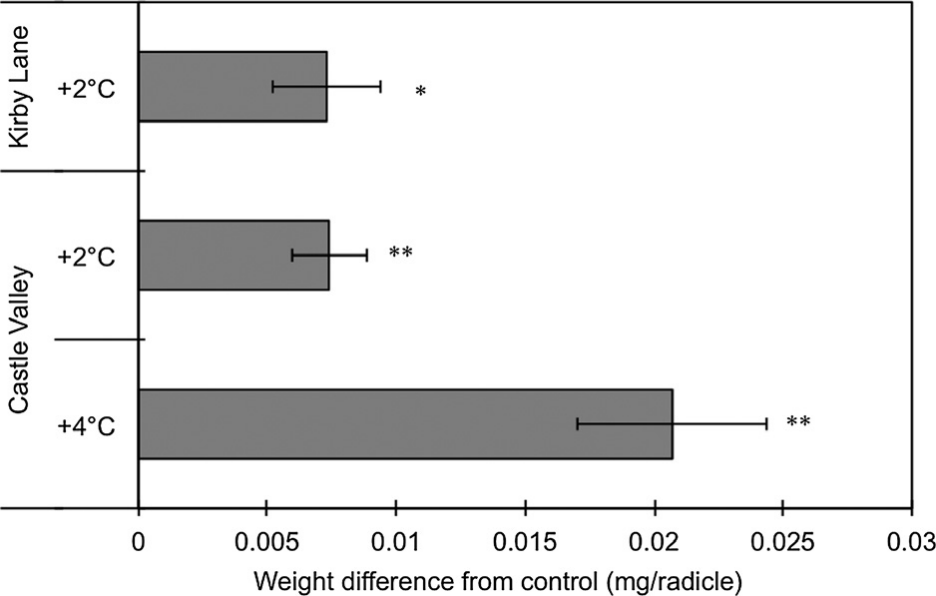
Weight difference between *B. tectorum* inflorescences collected from warmed and ambient plots in May 2010. * difference at α ≥ 0.05, ** difference at α ≥ 0.01.

Spikelets collected from +4°C plots at Castle Valley in 2010 weighed 4× more (mean weight 0.028 ± 0.004) than those from ambient plots (mean weight 0.007 ±0.001; df = 1, *F* = 28.29, *P* < 0.0001) and were 2× heavier in +2°C plots (mean weight 0.016 ± 0.002; df = 1, *F* = 10.60, *P* = 0.003; Fig. 6). In contrast, N concentrations were significantly higher in ambient plots (*t* = 2.28, df = 1, *P* = 0.03). We saw no other differences in C and N concentrations or C:N ratios. At Kirby Lane, spikelets from warmed plots were also heavier (mean weight 0.022 ± 0.002 g) than from ambient plots (mean weight 0.015 ± 0.002; df = 1, *F* = 6.35, *P* = 0.02; Fig. 6). The weight of all spikelets collected in 2011 did not differ between warmed and ambient plots.

### Greenhouse study

**Figure 7:**
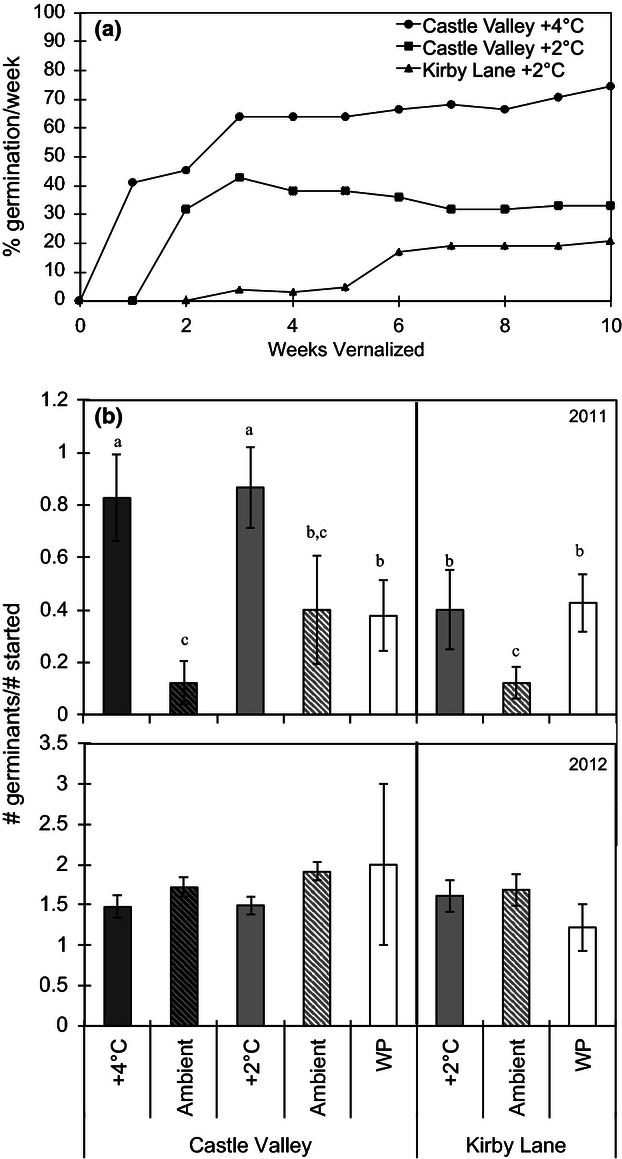
(a) Weekly percent germination difference between radicles started from warmed and control plots and (b) mean number (±SE) of germinated seedlings per radicle vernalized over winter in 2010–2011 and 2011–2012. Ambient represents plants collected from control plots associated with the warming treatments. WP represents “wild population” seeds collected from the general field site. CV is Castle Valley and KL is Kirby Lane. Different letters in (b) indicate statistically significant difference at α = 0.05. There were no significant differences in germination between seed sources in 2012.

Overall germination in 2010 from warmed seed sources was higher than from ambient seed sources for both warming treatments across both experimental sites. Within the first week, we saw 41% higher germination from +4°C plots and 32% higher germination from +2°C plots for Castle Valley plants, relative to the ambient plots within the same experimental block (Fig. 7a). By the end of the 10-week vernalization period, ambient seed sources within the +4°C produced four germinants and ambient seed sources within the +2°C blocks produced three germinants, in contrast to 11 germinants from +4°C plots, 13 from +2°C plots, and eight germinants from the Castle Valley WP. At Kirby Lane, germination rate was the same between the warmed and WP seed sources, which was 2× greater than from ambient seed sources (Fig. 7b). As a result, we saw 75% more germinants from +4°C seed sources, 31% more germinants from +2°C seed sources at Castle Valley, and 21% more germinants from warmed Kirby Lane seed sources (Fig. 7b). Offspring (F1) from warmed Castle Valley seed sources experienced low overall mortality (no mortality of seedlings from +4°C seed sources vs. 10% mortality from respective ambient seed sources and 1.6% mortality from +2°C seed sources vs. 19% mortality from respective ambient seed sources). The effects of warming on above and belowground biomass of the greenhouse plants were mixed. There were significant differences in reproductive output (number of inflorescences produced per plant) from F1 plants between seed sources from Castle Valley (*F*_4,37_ = 8.13, *P* = 0.0001, Tukey–Kramer q* = 2.88). Specifically, reproductive output was 5× lower from +4°C seed sources, relative to nonwarmed seed sources (1.2 spikelets ± 0.54 vs. 6.4 spikelets ± 1) and almost 2× greater from +2°C seed sources (4.4 spikelets ± 0.48 vs. 2.5 spikelets ± 0.86). Despite differences in reproductive output, we saw no differences in the weight of spikelets produced by F1 plants. Differences in root:shoot ratios (*F*_4,37_ = 3, *P* = 0.03; Tukey–Kramer q* = 2.88) were driven largely by differences in aboveground biomass (*F*_4,37_ = 3.74, *P* = 0.01; Tukey–Kramer q* = 2.88). Aboveground biomass of plants from warmed seed sources was greater than ambient, with the greatest biomass in offspring from +4°C seed sources. The root:shoot ratios were higher for offspring from +2°C seed sources than those from +4°C seed sources, but did not differ from the ambient F1 plants. In contrast to the results from Castle Valley, seedlings from warmed seed sources at Kirby Lane experienced greater mortality (24%) than seedlings from ambient seed sources (14%). We found no differences in F1 aboveground, root, or seed weight between warmed and ambient seed sources and no differences in reproductive output. We also found no differences in total C and N content between warmed and ambient plants and their offspring.

Seed germination was high across all treatments and plots in 2011. Despite no differences in germination, seedling mortality was lower from +2°C seed sources in Castle Valley (9.5% from +2°C seed sources vs. 40% from ambient seed sources). In contrast, seedling mortality was 2× higher for seedlings that germinated from +4°C plots (14.3% vs. 7.7% from nonwarmed seed sources). We observed no differences in germination or mortality for plants collected in 2011 from Kirby Lane. Similarly, we saw no differences in aboveground, root, or seed weight between plants from warmed and nonwarmed seed sources, no differences in growth rate, and no differences in reproductive output for offspring from Castle Valley and Kirby Lane.

### Variation at microsatellite loci

Of the 40 samples, 17 from the +4°C plots and 19 from the ambient plots amplified well at all or at least 4 of the 5 loci. Heterozygosity (across all 36 individuals) was 0.257, indicating that there is indeed genetic diversity at the Castle Valley field site. We found eight multilocus genotypes total, four common to both warmed and ambient plots, one found only in +4°C plots, and three found only in ambient plots. Despite this trend toward unique genotypes in the different plot types, there is little statistical evidence for genetic differentiation of warmed and ambient plots in these samples (*F*_ST_ is not significantly different from zero, *P* = 0.63).

## Discussion

Many recent studies report global shifts in the timing of important phenological events and have linked these shifts with changes in climate (Sherry et al. 2007; Bertin 2008). In our study, the effects of warming on phenology, biomass, and reproductive output were variable not only year to year, but also site to site. We found that the timing of phenological events, such as the timing of spring growth initiation, flowering, and summer senescence all advanced in warmed plots and the shift in phenology was progressively larger with greater warming. However, these large shifts in phenology and development were only evident following a wet winter and early spring. In the following two dry years, we saw no differences in phenology between warmed and ambient plots. In dryland ecosystems, moisture can be the most important factor influencing phenological development (Jolly and Running 2003) and the seasonal timing of precipitation has been shown to influence *B. tectorum* invasion across a range of sites (Bradford and Lauenroth 2006; Blumenthal et al. 2008). In our study, low wintertime precipitation overturned the positive effects of warming on plant phenology and development seen during wet years and combined with +4°C warming, had an overall negative effect.

Earlier spring growth and development in the warmed plots during the wet year was associated with increases in *B. tectorum* biomass and reproductive output on both soil types, likely the result of warmed soils enabling earlier and faster growth early in the spring, when water was not limiting. The overall extension of the growing season we observed in warmed plots was associated with taller, more fecund plants. The taller inflorescences from the warmed plots produced larger spikelets, suggesting that either the seeds were better provisioned or that the spikelets produced more seeds. Unfortunately, because we could not destructively harvest seeds from the spikelets, we were unable to get direct measures of seed production. However, the increase in spikelet size dramatically increased germination, offspring performance, and survival, all of which were progressively greater in the +4°C plots.

The differences between the germination rate from the site *B. tectorum* population (WP) and the ambient seed sources within the +4°C block in Castle Valley and ambient seed sources from Kirby Lane are puzzling and likely reflect site-specific mechanisms. In Castle Valley, the depressed germination from the ambient plots may reflect the legacy effects from the work on these plots in 2005–2008. During this period, the focus of the research within these plots was on biological soil crusts and a portion of each plot was destructively harvested for soil analyses (Zelikova et al. 2012) and the overall effect of destructive harvests likely remains, though we would expect a similar trend in both warmed and ambient plots. Another nonmutually exclusive possibility is that decreased germination from ambient seed sources may reflect the spatial heterogeneity of resource distribution at both sites. Regardless, there is no clear explanation for the discrepancy between germination from ambient and WP seed sources in 2011. However, these differences did not emerge in 2012, suggesting a differential ability to take advantage of increased soil water availability in 2011 between experimental and wild populations.

Whereas, the effect of warming on phenology, biomass, and reproductive output in the wet year of 2010 was straightforward across both sites, the effect of warming in drier years (2009, 2011, 2012) was more nuanced and influenced by both the amount of warming and site, likely differences in soil type. Overall, warming decreased plant density, size, and reproductive output in the +4°C plots in Castle Valley and the +2°C plots at Kirby Lane, but not the +2°C Castle Valley plots. Both the lack of an effect of warming in moderately dry years and the negative response to increasing temperature in the driest year support the growing body of work that suggests that plant responses to warming are tempered by soil moisture availability (Rustad et al. 2001; Belote et al. 2004). The fact that the negative effects of warming were only evident with +4°C warming and not with +2°C warming in Castle Valley also suggests a possible threshold response at that site. When compared with the significant effects of +2°C warming at Kirby Lane, the data also suggest an interacting effect of site, which may be related to soil texture. In comparison with the fine textured sandy loam soils in Castle Valley, the Kirby Lane site is characterized by well-drained sandy soils. Sandy soils generally have lower volumetric water content than loamy soils and thus lower plant water availability (Miller et al. 2006). It is possible that heating the less water limited, finer-textured loamy soils in Castle Valley 4°C was equivalent to heating the more water-limited sandy soils 2°C at Kirby Lane in terms of soil moisture loss and the effect on *B. tectorum*.

Other studies have shown an effect of soil type on *B. tectorum* growth at other sites in southeastern Utah (Miller et al. 2006). Miller et al. (2006) showed that soil water content was lowest in the sandy soils and *B. tectorum* fall germination and subsequent growth were also lower. Additionally, water limitation reduced fall germination and spring growth, indicating that *B. tectorum* establishment and success were both a function of two interrelated factors: soil texture and soil water availability. Thus, one potential explanation for the differences in plant responses to warming between experimental sites is that heating treatments depend on soil water content and heating sandy Kirby Lane soils by even 2°C may accelerate the loss of water from these already low moisture soils to achieve essentially the same effect as greater heating of fine textured Castle Valley soils. However, we cannot rule out that other differences between the Castle Valley and Kirby Lane sites, including differences in *B. tectorum* genotype, the structure and composition of the plant community, soil biota, and climate also contribute to the site-specific patterns we see.

In addition to the phenotypic responses we saw in the field, *B. tectorum* may also be capable of evolving in response to changing climate conditions. Evolutionary change can occur quickly, even under field conditions (Agrawal et al. 2012) and the potential to respond to warming (Willis et al. 2008), along with relatively rapid evolution (Busswell et al. 2011; Shaw and Etterson 2012), has been shown to be more common with introduced plant species than native species. In addition, annuals are expected to be more responsive to environmental changes than perennial grasses and shrubs (Franks et al. 2004). The traits that tend to be under selection include dispersal ability (Cheptou et al. 2008), size (Bossdorf et al. 2004), reproductive output (Ridley and Ellstrand 2009), and phenotypic plasticity (Richards et al. 2006) and when we observed changes in these traits under field conditions, they were associated with similar changes in the follow-up greenhouse experiments. It is not yet possible to know the relative contributions of maternal effects (which are likely to be strong) and genetic shifts in plants from our experimental plots. However, the existence of substantial genetic variation in North American *B. tectorum* populations (Rice and Mack 1991a,b,c; Leger et al. 2009; Scott et al. 2010; Huttanus et al. 2011) the documentation of local adaptation (Leger et al. 2009) at Nevada sites, and our evidence that even at the relatively small scale of our plots, there exists a diversity of genotypes, together suggest that adaptation to our experimental warming may be possible. Future tests of adaptation to experimental warming may thus help inform process-based modeling predictions of *B. tectorum* range shifts with ongoing climate change (Bradley et al. 2010).

In contrast to models that predict negative responses to warmer climate for *B. tectorum* in the southern part of its distribution (Bradford and Lauenroth 2006; Bradley 2009; Bradley et al. 2009), our results suggest that the invasive grass *B. tectorum* has the potential to quickly shift phenological development to earlier in the spring, with important consequences for growth and reproduction in wetter years. Importantly, these changes affect not only the plants that are directly experiencing the warmer conditions, but also their offspring. In fact, the larger seeds produced by warmed plants in the field have higher germination rates and yield larger offspring, even under greenhouse conditions with the warming treatment removed. The changes to *B. tectorum* phenology, biomass, and reproduction we observed in the field experiment translated to changes in offspring development both in the wet and dry years, suggesting the potential for evolutionary change. At this time, we cannot separate purely ecological responses from evolutionary ones, but earlier phenology is likely to be adaptive (Franks et al. 2004; Verdú and Traveset 2005; Kulpa and Leger 2013) and traits that affect plant fitness, such as reproductive output, are expected to respond to selection (Leimu and Fischer 2008; Kulpa and Leger 2013).

Shifts in the phenology of one species, especially an invasive, can have large consequences for other community members (Goergen et al. 2011), especially during the reproductive phases, when resource demand is generally high. The invasive *B. tectorum* has higher water use (Booth et al. 2003) and N uptake (DeFalco et al. 2003) than native grasses. Therefore, the earlier growth initiation we observed during a wet year likely allowed *B. tectorum* to access limited resources. The widespread *B. tectorum* invasion has already been shown to exert a selective pressure on native plant populations (Goergen et al. 2011) and how native plants cope with this pressure appears to be context-dependent. For native perennial grasses on the Colorado Plateau, the ability to shift phenology to earlier in the spring, when resources are less limiting, may be an effective competition strategy (Goergen et al. 2011). An alternative nonmutually exclusive possibility is tolerance of *B. tectorum* presence, especially during drier years. Long-term trends on the Colorado Plateau indicate that native perennial grasses are declining as a result of increasing annual temperatures (Munson et al. 2011), but *B. tectorum* appears to decline following years with below-average wintertime precipitation (Belnap and Phillips 2001). Such years may provide native grasses with the opportunity to overcome the negative effects of *B. tectorum* if they can persist while *B. tectorum* declines.

The southwestern United States is expected to become much drier by the end of this century (Cayan et al. 2010). Overall, our results highlight the importance of considering the interacting effects of temperature, precipitation, and site-specific characteristics such as soil texture on plant demography (Leuzinger et al. 2011) in predicting the effects of climate change. The interaction between natural climate variation and experimental warming we report may also help explain the lack of consistent effects of warming in other studies (Rustad et al. 2001) and highlights the need for longer-term studies to capture the natural variation in climate. Future modeling efforts to predict the spread of *B. tectorum* may benefit from taking a more mechanistic approach that incorporates both changes in plant phenology and demography as well as wintertime precipitation patterns. In addition, climate change experiments conducted across elevation or latitudinal gradients evaluate whether manipulated conditions select for particular genotypes or traits and provide the experimental context for understanding climate tracking and disequilibrium dynamics, which help estimate how quickly organisms can exploit newly available habitats. These combined approaches can improve future predictive distribution models (Ibáñez et al. 2013).

## References

[b1] Agrawal AA, Hastings AP, Johnson MTJ, Maron JL, Salminen JP (2012). Insect herbivores drive real-time ecological and evolutionary change in plant populations. Science.

[b2] Allen PS, Meyer SE (2002). Ecology and ecological genetics of seed dormancy in downy brome. Weed Sci.

[b3] Archer SR, Predick KI (2008). Climate change and ecosystems of the southwestern United States. Rangelands.

[b4] Badeck FW, Bondeau A, Böttcher K, Doktor D, Lucht W, Schaber J (2004). Responses of spring phenology to climate change. New Phytol.

[b5] Belnap J, Phillips SL (2001). Soil biota in an ungrazed grassland: response to annual grass (*Bromus tectorum*) invasion. Ecol. Appl.

[b6] Belote TR, Weltzin FJ, Norby RJ (2004). Response of an understory plant community to elevated [CO_2_] depends on differential responses of dominant invasive species and is mediated by soil water availability. New Phytol.

[b7] Bertin RI (2008). Plant phenology and distribution in relation to recent climate change. J. Torrey Bot. Soc.

[b8] Blumenthal D, Chimner RA, Welker JM, Morgan JA (2008). Increased snow facilitated plant invasion in mixedgrass prairie. New Phytol.

[b9] Booth MS, Caldwell MM, Start JM (2003). Overlapping resource use in three Great Basin species: implications for community invasibility and vegetation dynamics. J. Ecol.

[b10] Bossdorf O, Prati D, Auge H, Schmid B (2004). Reduced competitive ability of an invasive plant. Ecol. Lett.

[b11] Bradford JB, Lauenroth WK (2006). Controls over invasion of *Bromus tectorum*: the importance of climate, soil disturbance, and seed availability. J. Veg. Sci.

[b12] Bradley BA (2009). Regional analysis of the impacts of climate change on cheatgrass invasion shows potential risk and opportunities. Glob. Change Biol.

[b13] Bradley BA, Oppenheimer M, Wilcove DS (2009). Climate change and plant invasions: restoration opportunities ahead?. Glob. Change Biol.

[b14] Bradley BA, Wilcove DS, Oppenheimer M (2010). Climate change increases risk of plant invasion in the Eastern United States. Biol. Invasions.

[b15] Busswell JM, Moles AT, Hartley S (2011). Is rapid evolution common in introduced plant species?. J. Ecol.

[b16] Cayan DR, Das T, Pierce DW, Barnett TP, Tyree M, Gershunov A (2010). Future dryness in the southwest US and the hydrology of the 21st century drought. Proc. Natl. Acad. Sci. USA.

[b17] Cheptou PO, Carrue P, Rouifed S, Cantarel A (2008). Rapid evolution of seed dispersal in an urban environment in the weed Crepis sancta. Proc. Natl. Acad. Sci. USA.

[b18] Cleland EE, Chuine I, Menzel A, Mooney HA, Schwartz MD (2007). Shifting plant phenology in response to global change. Trends Ecol. Evol.

[b19] DeFalco LA, Bryla DR, Smith-Longozo V, Nowak RS (2003). Are Mojave Desert annual species equal? Resource acquisition and allocation for the invasive grass *Bromus madritensis* subsp. *rubens* (Poaceae) and two native species. Am. J. Bot.

[b20] Di'Tomaso JM (2000). Invasive weeds in rangelands: species, impacts, and management. Weed Sci.

[b21] Dukes JS, Mooney HA (1999). Does global change increase the success of biological invaders?. Trends Ecol. Evol.

[b22] Franks SJ, Sim S, Weis AE (2004). Rapid evolution of flowering time by an annual plant in response to a climate fluctuation. Proc. Natl. Acad. Sci. USA.

[b23] Goergen EM, Leger EA, Espeland EK (2011). Native perennial grasses show evolutionary responses to *Bromus tectorum* (cheatgrass) invasion. PLoS ONE.

[b24] Harte J, Torn MS, Chang F, Feifarek B, Kinzig AP, Shaw R (1995). Global warming and soil microclimate: results from a meadow-warming experiment. Ecol. Appl.

[b25] Hellmann JJ, Bryers JE, Bierwagen BG, Dukes JS (2008). Five potential consequences of climate change for invasive species. Conserv. Biol.

[b26] Huttanus TD, Mack RN, Novak SJ (2011). Propagule pressure and introduction pathways of *Bromus tectorum* (cheatgrass; poaceae) in the central United States. Int. J. Plant Sci.

[b27] Ibáñez I, Gornish ES, Buckley L, Debinski DM, Hellmann J, Helmuth B (2013). Moving forward in global-change ecology: capitalizing on natural variability. Ecol. Evol.

[b28] Jolly WM, Running SW (2003). Effects of precipitation and soil water potential on drought deciduous phenology in the Kalahari. Glob. Change Biol.

[b29] Kao RH, Brown CS, Hufbauer RA (2008). High phenotypic and molecular variation in Downy Brome (*Bromus tectorum*. Invasive Plant Sci. Manag.

[b30] Keeling CD, Chin JFS, Whorf TP (1996). Increased activity of northern vegetation inferred from atmospheric CO2 measurements. Nature.

[b31] Kimball BA (2005). Theory and performance of an infrared heater for ecosystem warming. Glob. Change Biol.

[b32] Kulpa SM, Leger EA (2013). Strong natural selection during plant restoration favors an unexpected suite of plant traits. Evol. Appl.

[b33] Leger EA, Espeland EK, Merrill KR, Meyer SE (2009). Genetic variation and local adaptation at a cheatgrass (*Bromus tectorum*) invasion edge in western Nevada. Mol. Ecol.

[b34] Leimu R, Fischer M (2008). A meta-analysis of local adaptation in plants. PLoS ONE.

[b35] Leuzinger S, Luo Y, Beier C, Dieleman W, Vicca S, Körner C (2011). Do global change experiments overestimate impacts on terrestrial ecosystems?. Trends Ecol. Evol.

[b36] Lin G, Phillips SL, Ehleringer JR (1996). Monosoonal precipitation responses of shrubs in a cold desert community on the Colorado Plateau. Oecologia.

[b37] Mack R (1981). Invasion of *Bromus tectorum* L. into western North America: an ecological chronicle. Agro-ecosystems.

[b38] McKone MJ (1985). Reproductive biology of several Bromegraasses (Bromus): breeding system, pattern of fruit maturation, and seed set. Am. J. Bot.

[b39] Meirmans PG, Van Tienderen PH (2004). GENOTYPE and GENODIVE: two programs for the analysis of genetic diversity of asexual organisms. Mol. Ecol. Notes.

[b40] Menzel A, Sparks TH, Estrella N, Koch E, Aasa A, Ahas R (2006). European phenological response to climate change matches the warming pattern. Glob. Change Biol.

[b41] Miller ME, Belnap J, Beatty SW, Reynolds RL (2006). Performance of *Bromus tectorum* L. in relation to soil properties, water additions, and chemical amendments in calcareous soils in southeastern Utah, USA. Plant Soil.

[b42] Moore PD (2004). Plant ecology: favoured aliens for the future. Nature.

[b43] Munson SM, Belnap J, Okin GS (2011). Responses of wind erosion to climate-induced vegetation changes on the Colorado Plateau. Proc. Natl. Acad. Sci. U S A.

[b44] Myneni RB, Keeling CD, Tucker CJ, Asrar G, Nemani RR (1997). Increased plant growth in the northern high latitudes from 1981 to 1991. Nature.

[b45] [NAST] National Assessment Synthesis Team, U.S. Global Change Research Program (2000). Climate Change Impacts on the United States: the Potential Consequences of Climate Variability and Change.

[b46] Parmesan C (2006). Ecological and evolutionary responses to recent climate change. Annu. Rev. Ecol. Evol. Syst.

[b47] Parmesan C, Yohe G (2003). A globally coherent fingerprint of climate change impacts across natural systems. Nature.

[b48] Partanen J, Koski V, Hänninen H (1998). Effects of photoperiod and temperature on the timing of bud burst in Norway spruce (*Picea abies*. Tree Physiol.

[b49] Ramakrishnan AP, Coleman CE, Meyer SE, Fairbanks DJ (2002). Microsatellite markers for *Bromus tectorum* (cheatgrass). Mol. Ecol. Notes.

[b50] Rice KJ, Mack RN (1991a). Ecological genetics of *Bromus tectorum* I: a hierarchical analysis of phenotypic variation. Oecologia.

[b51] Rice KJ, Mack RN (1991b). Ecological genetics of *Bromus tectorum* II: intraspecific variation of phenotypic plasticity. Oecologia.

[b52] Rice KJ, Mack RN (1991c). Ecological genetics of *Bromus tectorum* III: the demography of reciprocally sown populations. Oecologia.

[b53] Richards CL, Bossdorf O, Muth NZ, Gurevitch J, Pigliucci M (2006). Jack of all trades, master of some? On the role of phenotypic plasticity in plant invasions. Ecol. Lett.

[b54] Ridley CE, Ellstrand NC (2009). Evolution of enhanced reproduction in the hybrid-derived invasive, California wild radish (*Raphanus sativus*. Biol. Invasions.

[b55] Roundy BA, Hardegree SP, Chambers JC, Whittaker A (2007). Prediction of cheatgrass field germination potential using wet thermal accumulation. Rangeland Ecol. Manage.

[b56] Rustad LE, Campbell JL, Marion GM, Norby RJ, Mitchell MJ, Hartley AE (2001). A meta-analysis of the response of soil respiration, net nitrogen mineralization, and aboveground plant growth to experimental ecosystem warming. Oecologia.

[b57] Schwinning S, Starr BI, Ehleringer JR (2005a). Summer and winter drought in a cold desert ecosystem (Colorado Plateau) part I: effects on soil water and plant water uptake. J. Arid Environ.

[b58] Schwinning S, Starr BI, Ehleringer JR (2005b). Summer and winter drought in a cold desert ecosystem (Colorado Plateau) part II: effects on plant carbon assimilation and growth. J. Arid Environ.

[b59] Schwinning S, Belnap J, Bowling DR, Ehleringer JR (2008). Sensitivity of the Colorado Plateau to change: climate, ecosystems, and society. Ecol. Soc.

[b60] Scott JW, Meyer SE, Merrill KR, Anderson VJ (2010). Local population differentiation in *Bromus tectorum* L. in relation to habitat-specific selection regimes. Evol. Ecol.

[b61] Seager R, Vecchi DA (2010). Greenhouse warming and the 21^st^ century hydroclimate of southwestern North America. Proc. Natl. Acad. Sci. USA.

[b62] Seager R, Ting M, Held I, Kushnir Y, Lu J, Vecchi G (2007). Model projections of an imminent transition to a more arid climate in southwestern North America. Science.

[b63] Shaw RG, Etterson JR (2012). Rapid climate change and the rate of adaptation: insight from experimental quantitative genetics. New Phytol.

[b64] Sherry RA, Zhou X, Gu S, Arnone JA, Schimel DS, Verburg PS (2007). Divergence of reproductive phenology under climate warming. Proc. Natl. Acad. Sci. USA.

[b65] Smith SD, Huxman TE, Zitzer SF, Charlet TN, Housman DC, Coleman JS (2000). Elevated CO_2_ increases productivity and invasive species success in an arid ecosystem. Nature.

[b66] Smith SJ, Thomson AM, Rosenberg NJ, Izaurralde RC, Brown RA, Wigley TML (2005). Climate change impacts for the conterminous USA: an integrated assessment. Part I. Scenarios and Context. Clim. Change.

[b67] Thuiller W, Richardson DM, Pysek P, Midgley GH, Hughes GO, Rouget M (2005). Niche-based modeling as a tool for predicting the risk of alien plant invasion at the global scale. Glob. Change Biol.

[b68] Verdú M, Traveset A (2005). Early emergence enhances plant fitness: a phylogenetically controlled meta-analysis. Ecology.

[b69] Walther G, Roques A, Hulme PE, Sykes MT, Pysek P, Kühn I (2009). Alien species in a warmer world: risks and opportunities. Trends Ecol. Evol.

[b70] Wein NE, West RW (1971). Phenology of salt desert plants near contour furrows. J. Range Manag.

[b71] Weltzin JF, Belote RT, Sanders NJ (2003). Biological invaders in a greenhouse world: will elevated CO_2_ fuel plant invasions?. Front. Ecol. Environ.

[b72] Willis CG, Ruhfel B, Primack RB, Miller-Rushing AJ, Davis CC (2008). Phylogenetic patterns of species loss in Threau's woods are driven by climate change. Proc. Natl. Acad. Sci. U S A.

[b73] Wolkovich EM, Cook BI, Crimmins JM, Betancourt JL, Travers SE, Pau S (2012). Warming experiments underpredict plant phonological responses to climate change. Nature.

[b74] Zelikova TJ, Housman DC, Grote EE, Neher DA, Belnap J (2012). Warming and increased precipitation frequency on the Colorado Plateau: implications for biological soil crusts and soil processes. Plant Soil.

[b75] Zhang R, Jongejans E, Shea K (2011). Warming increases the spread of an invasive thistle. PLoS ONE.

[b76] Zinka LH (2003). Evaluation of the growth response of six invasive species to past, present and future atmospheric carbon dioxide. J. Exp. Bot.

